# Vitamin D status in Kancheepuram District, Tamil Nadu, India

**DOI:** 10.1186/s12889-018-6244-5

**Published:** 2018-12-05

**Authors:** John Mechenro, Giriprasad Venugopal, M. Buvnesh Kumar, D. Balakrishnan, Balakrishnan S. Ramakrishna

**Affiliations:** 1 SRM Institute of Gastroenterology, Hepatobiliary Sciences & Transplantation, SIMS Hospital, Jawaharlal Nehru Road, Vadapalani, Chennai, Tamil Nadu India; 2 Department of Medical Research, SRM Medical College Hospital & Research Centre, SRM University, Kancheepuram District, Kattankulathur, Tamil Nadu India; 3 Department of Community Medicine, SRM Medical College Hospital & Research Centre, SRM University, Kancheepuram District, Kattankulathur, Tamil Nadu India; 4 Department of Community Medicine, Chettinad Hospital & Research Institute, Kelambakkam, Tamil Nadu India

**Keywords:** Vitamin D, Deficiency, Insufficiency, Sun exposure, Epidemiology, India

## Abstract

**Background:**

Vitamin D has multifarious roles in maintenance of health and prevention of disease. The present study was undertaken to assess the vitamin D status of a rural adult south Indian population and to identify its associations with socioeconomic status and cultural practices.

**Methods:**

Between June 2015 and July 2016, 424 healthy adults residing in Kattankulathur block in Tamil Nadu, India, provided venous blood samples and answered questions by personal interview. 25-hydroxy vitamin D was estimated by ELISA.

**Results:**

Fifty nine (13.9%) of the 424 participants had 25OHD levels below 12 ng/mL (vitamin D deficient) and 175 (41.3%) had 25OHD levels between 12 to 20 ng/mL (vitamin D insufficiency). In univariate analysis, demographic factors associated with vitamin D status included education, occupation, socioeconomic class, and birthplace; lifestyle factors included sun exposure time, skin surface exposed to sunlight, use of sunscreen, awareness of vitamin D, and consumption of fish; and hygiene related factors included source of drinking water, availability of tap water at home, and closed toilet at home. In ordinal logistic regression, the following variables were found to be independently associated with vitamin D sufficiency: Duration of daily sun exposure below 30 min (Odds ratio 0.31, 95% confidence intervals 0.14–0.71, *P* = 0.006), sun exposure 30–60 min (OR 0.49, 95% CI 0.30–0.80, *P* = 0.004), male gender (OR 2.00, 95% CI 1.30–3.09, *P* = 0.002), higher level of education (OR 0.80, 95% CI 0.69–0.94, *P* = 0.005), non-consumption of fatty fish (OR 0.48, 95% CI 0.24–0.85, *P* = 0.035) and presence of closed toilet system at home (OR 0.59, 95% CI 0.37–0.93).

**Conclusion:**

VDD and VDI are highly prevalent in this rural Indian community. The study identifies socioeconomic and behavior patterns that negatively impact vitamin D sufficiency, thus providing a basis for targeted intervention.

**Electronic supplementary material:**

The online version of this article (10.1186/s12889-018-6244-5) contains supplementary material, which is available to authorized users.

## Background

Vitamin D deficiency (VDD) is very prevalent in many parts of the world [1]. The major cause of VDD is lack of adequate exposure to sunlight due to various lifestyle factors and practices [1]. The amount of sunlight exposure needed to sustain adequate vitamin D levels varies with the skin colour of the individual. For white Caucasians it has been estimated that exposing the face, hands, forearms and legs to sunlight for 9 min daily during lunchtime in the UK summer would ensure vitamin D sufficiency [2], whereas an individual with brown skin would require a 25 min exposure under the same conditions [3]. Vitamin D can be obtained from dietary sources but few foods naturally contain vitamin D. Foods that are fortified with vitamin D are often not consumed in quantities sufficient to meet the body’s vitamin D requirement [1]. Overt VDD is linked to bone disease such as rickets in the very young and osteomalacia in adults. In addition, lesser degrees of VDD are associated with an increased risk of diseases as diverse as cancer, diabetes, skin disease and neuropsychiatric disease [4–7], thus making VDD a problem of public health importance.

Vitamin D3 or cholecalciferol is produced in the skin from 7-dehydrocholesterol through the action of ultraviolet (UVB) rays from the sun. Both UVB intensity and skin pigmentation contribute to the rate of D3 formation [8]. Melanin in the skin blocks UVB from reaching 7-dehydrocholesterol as do clothing and sun-screen. The intensity of UVB from sunlight varies according to season and latitude, being maximal close to the equator. Vitamin D2 or ergocalciferol can be obtained from plants and fungi, principally mushrooms, if they have been exposed to UVB. Vitamin D3 and D2 undergo 25-hydroxylation in the liver to form 25-hydroxy vitamin D (25OHD), and this further undergoes 1α hydroxylation in the kidney to become the active form of vitamin D [8]. The plasma level of 25OHD, (which is the long-lived circulating metabolite of vitamin D) is used most commonly as the marker of an individual’s vitamin D status. It has been shown that the gut microbiota have the capacity to regulate 25 hydroxylation of vitamin D in the liver [9], thus influencing circulating 25OHD concentration.

Rickets in children is associated with circulating concentrations of 25OHD of less than 20 ng/mL (50 nmol/L) [10], as is osteomalacia in adults [11]. Circulating levels of 25OHD of less than 20 ng/mL have been advocated as a cut-off to define VDD [12], ,but the more restrictive Institute of Medicine recommendations suggest the use of circulating 250HD levels less than 12 ng/mL to define VDD, and levels between 12 and 20 ng/mL to define vitamin D insufficiency (VDI) [13]. In the present study, we use the more restrictive definition of VDD.

Social, economic and cultural factors determine an individual’s access to resources, including material goods, healthcare, and educational opportunities. Population health and nutritional status is influenced by socioeconomic status and other social determinants, and these are used in risk predictive modeling of disease [14, 15]. In India, the Kuppuswamy scale and its modifications are used to classify the socioeconomic status of a family using a composite score of education and occupation of the head of the family along with monthly income of the family [16].

Vitamin D status is poor in many countries, with less than 50% of people worldwide being vitamin D sufficient. Deficiency is more common in the Middle East, China, Mongolia and India [17]. It is reported that only about 20% of healthy Indians have vitamin D sufficiency [18, 19], and this is a matter of particular concern given that the country has adequate sunshine. This may indicate a major role for social and cultural factors in the determination of an individual’s vitamin D status.

The present study was undertaken to assess the vitamin D status of a rural adult south Indian population and to identify its associations with socioeconomic and cultural factors.

## Methods

### Study area and study design

This cross-sectional study was conducted in the Kattankulathur (latitude: 12° 81′ 10”N, 80° 03′ 05″E) block of Kancheepuram district in Tamil Nadu, India. The block comprises 39 villages with 133 habitations with a total population of 213,850 as enumerated in 2012–2013. Study participants were apparently healthy adult men and women living in these villages. The inclusion criteria for the study were that individuals should be healthy, aged 18 years or more, residing in the study area for at least 1 year prior to the study, and willing to provide written consent to participate in the study. Those with self-reported coronary artery disease, arthritis, or cancer were excluded. The study was undertaken over a 14 month period from June 2015 to July 2016. A modified 30 × 7 sampling method was used [20, 21]. Assuming a community VDD prevalence of 50% (a conservative estimate derived from published data from India [19]) a sample size of 420 was calculated. The sample size calculation formula was z^2^*p*q/d^2^ where the standardized score for 95% confidence level (Z) =1.96, *p* = 0.5, q = 1-p = 0.5 & d = 0.5, to estimate a VDD prevalence of 50% with a ± 5% error margin. Using a 30-cluster sampling, the sample size was calculated as 14 adults per cluster or a total of 420 adults. Thirty of the 133 habitations in Kattankulathur were selected, and the houses to be sampled were chosen accordingly.

### Data collection and sampling

Data collection was undertaken between June to September 2015. Participants were selected in the study area as follows. The first participating household was selected using the Expanded Programme on Immunization (EPI) recommendation [20], i.e., the centre of the area corresponding to the selected habitation was reached and a direction was selected after spinning a bottle and following the direction in which the cap pointed. The first house in that direction was selected as the first household and the first eligible adult encountered in that house was recruited. Subsequent households were selected by following the EPI strategy of going to the household whose door was nearest to the current household until 14 eligible participants accrued in that habitation.

An interview schedule was created. Informed written consent was obtained from each of the participants. Each participant was interviewed directly by the first author using a predesigned form (Additional file 1). Data was collected on demographic profile and socioeconomic status (age, sex, marital status, education, income, occupation, and the following life style factors: Duration of sun exposure, body surface area exposed to sunlight, use of sunscreen while going outdoors, intake of fatty fish, beef, liver and milk. Participants were asked to recall the average duration spent outdoors during weekdays and the weekend in the past 1 month and this was categorized as 15–30 min, 30–60 min, 1–2 h and > 2 h per day [22]. The body surface exposed to sunlight when going outdoors was categorized as fully covered (only hands and feet exposed), wearing short sleeves (exposing hands, arms and forearms), wearing short sleeved T-shirt and shorts or dhoti (a single cloth tied around the waist and hitched up to expose the legs) or only wearing shorts or dhoti (which also exposed the torso) [22]. Data on average intake of fatty fish, beef, liver (from poultry or cattle) and milk were obtained by asking participants to recall their intake in the 2 weeks prior to interview. Data were also obtained on use of multivitamin supplements containing vitamin D, and general awareness about vitamin D. The latter was verified by asking whether the participant had heard of vitamin D, whether he or she had learnt anything about vitamin D in school, through television, or through promotional literature distributed by health organizations or in advertisements. In addition, we asked questions on birth (urban versus rural), source of drinking water for the household, use of boiled or bottled water at home, availability of tap water at home, and availability of a close toilet system at home. This latter set of questions as aimed at elucidating information related to hygiene factors that are believed to influence the composition of the gut microbiome of an individual. Data were assigned into categories for analysis. Socioeconomic status was assigned using a modified Kuppuswamy score which was calculated based on the level of education, occupation and income of the head of the household [16].

### Sample collection and vitamin D assay

Venous blood samples were collected at home from consenting participants using Vacutainers and were transported to the laboratory within an hour. Samples were immediately centrifuged to separate the serum which was stored at -20 °C. Total 25-OH vitamin D was measured in duplicate by ELISA using the DIAsource 25-OH Vitamin D Total kit (Catalog No. KAP1971, DIAsource Immunoassays SA, Louvain-la Neuve, Belgium), which uses monoclonal antibodies to measure 25-OH D2 and 25-OH D3 and has been certified by the Vitamin D External Quality Assessment Scheme. The intra-assay coefficient of variation (range 2.7–7.8%) and the inter-assay coefficient of variation (range 4.7–9.4%) for this assay were both less 10%. Appropriate controls and calibrators (provided with each kit) were used with each ELISA plate to generate standard curves. Based on the level, an individual was classified as vitamin D deficient if serum level was < 12 ng/mL, vitamin D insufficient if 12–20 ng/mL, and vitamin D replete or sufficient if > 20 [13]. The vitamin D estimation was done at the GI Research Lab, SIMS Hospitals, Vadapalani, Chennai, by an investigator who was blinded to the participant characteristics.

### Statistical analysis

Data were entered in MS-Excel and analyzed using IBM SPSS version 20. In univariate analysis, Pearson’s chi-square test was used to determine association of the variables with vitamin D status. *P* values < 0.05 were considered as statistically significant. Variables that were associated with vitamin D status on univariate analysis with P values less than 0.10 (this P value was selected in order to limit the number of variables in the model) were entered into an ordinal logistic regression analysis in which vitamin D status was the dependent variable with the ordinal categories ordered as deficient, insufficient, and sufficient. The independent variables introduced into the model as factors were sun exposure time per day, SES group, gender and age group, consumption of fish and consumption of milk; educational qualification, occupation, birth place, awareness of VDD, source of drinking water, boiled drinking water, tap water available at home and closed toilet system at home were included as covariates. There were many cells with small observed and predicted counts, and the goodness-of-fit statistic showed a Pearson Chi-square of 848.087 and Deviance Chi-square of 766.370 (df 807. Since there were cells with small observed counts, the overall model fitting test showed a − 2 log likelihood of 841.124 for intercept only compared to 772.151 for the final model, leading to a Chi-square of 68.973 (df 11) and a significance < 0.0001, indicating that the model with the predictors was valid. The proportional odds assumption of ordinal logistic regression analysis was tested using the test of parallel lines. The test showed a − 2 log likelihood of 752.48 for the null hypothesis model compared to 728.942 for the general model, leading to a Chi-square of 23.538 (df 19) and a significance of 0.214 thus affirming the null hypothesis that the proportional odds were the same across response categories. Odds ratios and 95% confidence intervals are presented for the variables that showed independent association with vitamin D status.

## Results

### Demographics

The total number of households approached for participation in the study was 1432, of which 432 individuals gave consent and were interviewed, and 424 gave blood samples for the study. Of the 424 participants, 179 (42%) were male. The mean (SD) age was 38.2 (16.3) years for the male participants and 42.5 (13.5) years for the female participants.

### Overall vitamin D status of the population

Overall mean (SD) 25OHD levels were 20.5 (9.3) ng/mL in the participants, and were 21.8 (10.7) among males compared to 19.7 (8.0) in women. The median value was 19.2 ng/mL and the range was 7. 6 to 100.8 ng/mL. Of the 424 participants, 190 had vitamin 25OHD levels higher than 20 ng/mL, indicating that only 44.8% of this adult population was vitamin D sufficient. One hundred and seventy five participants had vitamin D levels between 12 and 20 ng/mL (VDI) and 59 had levels less than 12 ng/ml (VDD).

### Association of vitamin D status with demographic factors

Demographic variables that were significantly associated with vitamin D status included the educational status of the individual, the occupational profile of the individual, the socioeconomic class, and birth in a rural or urban area. Gender, marital status, age, community, and religion did not show significant associations with vitamin D status (Table 1).

**Table 1:** Vitamin D status in relation to social and economic categories

Variables	Groups	*n*	Plasma 25OH vitamin D status			*P* value
			Deficient (< 12 ng/mL)	Insufficient (12–20 ng/mL)	Sufficient (> 20 ng/mL)	
Gender	Male	179	21 (11.7)	67 (37.4)	91 (50.8)	0.0963
	Female	245	38 (15.5)	108 (44.1)	99 (40.4)	
Marital status	Unmarried	82	16 (19.5)	32 (39)	34 (41.5)	0.2635
	Married / Widow	342	4312.6)	143 (41.8)	156 (45.6)	
SES Category	Upper + Upper Middle	76	12 (15.8)	38 (50)	26 (34.2)	0.0066
	Lower Middle	234	41 (17.5)	90 (38.5)	103 (44)	
	Upper Lower + Lower	114	6 (5.3)	47 (41.2)	61 (53.5)	
Age group (Years)	18–30	135	26 (19.3)	61 (45.2)	48 (35.6)	0.0942
	31–45	140	20 (14.3)	55 (39.3)	65 (46.4)	
	46–60	101	9 (8.9)	41 (40.6)	51 (50.5)	
	Above 60	48	4 (8.3)	18 (37.5)	26 (54.2)	
Education	Up to primary school	260	30 (11.5)	87 (33.5)	143 (55.0)	< 0.0001
	High school and above	164	29 (17.7)	88 (53.7)	47 (28.6)	
Occupation	Non-professional	411	53 (12.9)	172 (41.8)	186 (45.3)	0.0029
	Professional	13	6 (46.2)	3 (23.1)	4 (30.8)	
Birth Place	Rural	393	49 (12.5)	164 (41.7)	180 (45.8)	0.0085
	Urban	31	10 (32.3)	11 (35.5)	10 (32.3)	
Religion	Hindu	353	46 (13)	143 (40.5)	164 (46.5)	
	Christian	62	11 (17.7)	28 (45.2)	23 (37.1)	0.5751
	Muslim	9	2 (22.2)	4 (44.4)	3 (33.3)	

### Association of vitamin D status with lifestyle factors

Exposure to sunlight is the major natural source of Vitamin D, and varied from 15 min per day to > 120 min per day. Sun exposure time per day, portions of the body exposed to sunlight, usage of sunscreen, and awareness of vitamin D, were all significantly associated with vitamin D status in univariate analysis. Only 16 individuals (3.8% of the surveyed population) used vitamin D and other vitamin supplements. Vitamin D status was not associated with usage of multivitamins containing vitamin D (Table 2).

**Table 2:** Association of Vitamin D status with life style factors

Variables	Groups	*n*	Plasma 25OH vitamin D status			*P* value
			Deficient (< 12 ng/mL)	Insufficient (12–20 ng/mL)	Sufficient (> 20 ng/mL)	
Sun exposure time per day	15–30 min	29	10 (34.5)	12 (41.4)	7 (24.1)	0.0001
	30–60 min	137	27 (19.7)	68 (49.6)	42 (30.7)	
	60–120 min	84	9 (10.7)	36 (42.9)	39 (46.4)	
	> 120 min	174	13 (7.5)	59 (33.9)	102 (58.6)	
Body portions exposed to sunlight	Face, upper and lower limbs exposed	86	10 (11.6)	26 (30.2)	50 (58.1)	0.0196
	Face and upper limbs exposed	338	49 (14.5)	149 (44.1)	140 (41.4)	
Usage of sun screen	No	408	55 (13.5)	164 (40.2)	189 (46.3)	0.0066
	Yes	16	4 (25)	11 (68.8)	1 (6.3)	
Usage of vitamin D supplements	No	408	58 (14.2)	170 (41.7)	180 (44.1)	0.3218
	Yes	16	1 (6.3)	5 (31.3)	10 (62.5)	
Awareness of vitamin D	No	356	48 (13.5)	137 (38.5)	171 (48)	0.0081
	Yes	68	11 (16.2)	38 (55.9)	19 (27.9)	

### Association of vitamin D status with consumption of specific foods

Consumption of fish was associated with vitamin D status, with vitamin D sufficiency in 27.8% of those who did not consume fish compared to 46.4% of those who ate fish (Table 3). Food habits that showed no association with vitamin D status included vegetarianism, consumption of liver, consumption of beef, and frequent consumption of milk.

**Table 3:** Association of Vitamin D status with dietary habits. None of the foods was fortified with vitamin D

Variables	Groups	n	Plasma 25OH vitamin D status			*P* value
			Deficient (< 12 ng/mL)	Insufficient (12–20 ng/mL)	Sufficient (> 20 ng/mL)	
Consumption of fatty fish	No	36	11 (30.6)	15 (41.7)	10 (27.8)	0.0056
	Yes	388	48 (12.4)	160 (41.2)	180 (46.4)	
Consumption of beef	No	249	36 (14.5)	108 (43.4)	105 (42.2)	0.4248
	Yes	175	23 (13.1)	67 (38.3)	85 (48.6)	
Consumption of liver	No	205	34 (16.6)	87 (42.4)	84 (41.)	0.1766
	Yes	219	25 (11.4)	88 (40.2)	106 (48.4)	
Consumption of milk	No	221	26 (11.8)	102 (46.2)	93 (42.1)	0.0835
	Yes	203	33 (16.3)	73 (36.0)	97 (47.8)	

### Association of vitamin D status with parameters of hygiene

Hygiene parameters were evaluated in view of their importance as determinants of public health and the gut microbiota in low and middle income countries. Vitamin D sufficiency was significantly less in individuals who habitually used bottled water compared to those who obtained their drinking water from a well, borewell or public tap (Table 4). Interestingly, other hygiene parameters that associated with vitamin D status included use of boiled or filtered drinking water, availability of tap water at home, and availability of a closed toilet system at home (Table 4).

**Table 4:** Association of Vitamin D status with hygiene related factors

Variables	Groups	*n*	Plasma 25OH vitamin D status			*P* value
			Deficient (< 12 ng/mL)	Insufficient (12–20 ng/mL)	Sufficient (> 20 ng/mL)	
Source of drinking water	Bore well	33	3 (9.1)	17 (51.5)	13 (39.4)	0.0001
	Well	19	2 (10.5)	9 (47.4)	8 (42.1)	
	Public tap	285	30 (10.5)	106 (37.2)	149 (52.3)	
	Bottled water	87	24 (27.6)	43 (49.4)	20 (23.0)	
Boiled/filtered drinking water	No	323	34 (10.5)	129 (39.9)	160 (49.5)	0.0001
	Yes	101	25 (24.8)	46 (45.5)	30 (29.7)	
Tap water at home	No	242	26 (10.7)	94 (38.8)	122 (50.4)	0.0121
	Yes	182	33 (18.1)	81 (44.5)	68 (37.4)	
Closed toilet system	No	235	24 (10.2)	83 (92)	128 (54.5)	0.0001
	Yes	189	35 (18.5)	92 (48.7)	62 (32.8)	

### Multivariable analysis

Ordinal logistic regression was done using vitamin D status as the dependent variable, in which VDD, VDI, and vitamin D sufficiency were arranged in ordinal fashion. Table 5 shows the factors that were significantly associated with vitamin D status and presents the estimate and direction of their effect on vitamin D sufficiency. Gender (females less likely to be sufficient), sunlight exposure (< 60 min pre day less likely to be sufficient), educational qualification (higher education less likely to be sufficient), consumption of fish (non-consumption less likely to be sufficient), and closed toilet system at home (closed toilet less likely to be sufficient). The following factors were not associated with vitamin D status: SES, occupation, age group, birth place, use of sunscreen, awareness of vitamin D deficiency, milk consumption, source of drinking water, use of boiled/filtered water, and availability of tap water at home.

**Table 5:** Ordinal regression analysis with vitamin D status as the dependent variable. Vitamin D status was classified as deficient, insufficient or sufficient. Odds ratios and 95% confidence intervals, with vitamin D sufficiency as the reference status, are shown. The category shown in parenthesis against each dichotomous variable is associated with the odds ratio of being vitamin D sufficient as compared to the alternative category

	Odds ratio	95% Confidence interval		Sig.
		Upper bound	Lower bound	
Birth place (Urban)	0.61	0.29	1.29	0.195
Awareness of Vitamin D (Yes)	1.14	0.66	2.00	0.631
Source of drinking water (Bottled)	0.86	0.66	1.12	0.265
Drinking water boiled/filtered – (Yes)	0.67	0.41	1.09	0.104
Tap water at home (Yes)	1.02	0.65	1.59	0.931
Closed toilet system (Yes)	0.59	0.37	0.93	0.022
Consumption of milk (Yes)	1.11	0.74	1.65	0.613
Educational qualification (High school and above)	0.80	0.69	0.94	0.005
Occupation (Professional)	1.01	0.91	1.13	0.802
Sun exposure per day 15–30 min	0.31	0.14	0.71	0.006
Sun exposure per day 30–60 min	0.49	0.30	0.80	0.004
Sun exposure per day 60–120 min	0.82	0.48	1.40	0.468
Sun exposure per day > 120 min	0^(a)^			.
SES Upper & Upper Middle Class	1.10	0.56	2.15	0.780
SES Lower Middle Class	0.71	0.44	1.15	0.166
SES Upper Lower & Lower Class	0^(a)^			.
Gender (Male)	2.00	1.30	3.09	0.002
Age group 18–30 years	0.76	0.36	1.58	0.462
Age group 31–45 years	1.03	0.51	2.09	0.924
Age group 46–60 years	1.35	0.65	2.82	0.423
Age group > 60 years	0(a)			.
Consumption of fatty fish (No)	0.48	0.24	0.95	0.035

^(a)^This parameter is set to zero because it is redundant

## Discussion

The present study, from a rural area of Tamil Nadu, indicates that only 44.8% of the population was sufficient in vitamin D. The study further identified that vitamin D status was independently associated with time to which the body was exposed to sunlight very day, the gender of the participant, the level of education of the individual, the consumption of fatty fish, and presence of a closed toilet system at home. Each of these associations is discussed at greater length.

Both VDD and VDI were more common in individuals who had studied beyond primary school. We could not find any description of a similar observation previously in Indian studies of vitamin D status. It is possible that those individuals with higher educational attainments were involved in occupations that allowed less exposure to sunlight. The lack of vitamin D fortification in foods consumed in this location probably contributed to this. While occupational status was associated with VDD and VDI in univariate analysis, it was not significantly associated with vitamin D status in the multivariable analysis. In Western countries, several studies have uniformly identified higher education as being associated with better vitamin D status [23–25], quite the opposite of what we observed in our study.

Exposure to sunshine is a major factor in generating vitamin D in the body and is important in maintaining sufficiency of the vitamin in the body. The time to which the body needs to be exposed to sunlight varies with skin color. Dark skin color as is common in south India requires a longer time of exposure to sunlight for generation of vitamin D. Although we did not formally assess skin pigmentation in the study population, almost all had Type 5 skin pigmentation based on the Fitzpatrick scale [26]. The melanin pigment in skin absorbs ultraviolet radiation and protects it from damage; however it also reduced the ultraviolet radiation necessary for vitamin D synthesis in the skin. Comparative studies in the United Kingdom have shown that white-skinned Caucasians required only 9 min of daily UK summer sunlight exposure of face, forearms and lower legs to meet their vitamin D needs, while those with brown skin required 25 min of daily sunlight exposure under similar conditions [1, 2]. Our participants had brown skin probably similar to the UK cohort in the second study above. Penetration of UVB radiation through the atmosphere is greater at midday than early in the morning. In addition to skin color and time of day, the use of clothing to cover the entire body and both upper and lower limbs and the use of sun screen were other cultural practices that were associated with VDD. In the multivariable analysis, sunlight exposure time less than 60 min per day negatively impacted vitamin D sufficiency, with 15–30 min exposure having a greater negative effect than 30–60 min exposure per day. Our study suggests that going outdoors in the sun for greater than 60 min per day is required for maintaining vitamin D sufficiency. A study conducted among urban men in Pune, India also suggested that more than 1 h of casual midday sunlight exposure was necessary to maintain vitamin D levels [27]. In the present study, exposure of face, arms and legs to sunlight was associated protectively with VDI compared to exposure of face and arms alone.

The higher prevalence of VDD in individuals drinking protected water such as bottled water or water from bore wells is an association that has not earlier been documented. This and the other variables shown in Table 4 have been used as measures of hygiene in cross sectional association studies, and hygiene is known to influence health outcomes by effects on the composition of the gut microbiota [28]. While the association between hygiene and vitamin D status may be due to chance it is interesting to consider that there may be a direct link between serum 25OHD levels and domestic hygiene. The vitamin D produced in the skin is inactive and undergoes its first hydroxylation (25 hydroxylation) in the liver. The gut microbiota, through fibroblast growth factor 23, regulate 25 hydroxylation of vitamin D in the liver [9]. In germ free mice, plasma 25OHD levels were low, andincreased when gut microbiota were introduced. Since we (and other investigators) used plasma 25OHD levels to define vitamin D status, it was reasonable to include variables relating to hygiene (which greatly influences the gut microbiome) in this study evaluating associations of vitamin D status.

Sufficiency of vitamin D is necessary not only for bone health but for a variety of other metabolic and immune processes including effects mediated through vitamin D receptors distributed ubiquitously in the body. In the present study we identify associations of lifestyle and cultural practices with vitamin D status. The newly identified interaction between circulating vitamin D, vitamin D receptors in the gut and the gut microbiome [29] underscores the emerging importance of vitamin D in human physiology and its role in the maintenance of health. It is very likely that poor or marginal vitamin D nutrition is an important determinant of ill health at both population and individual level in this population.

Limitations of the present study include that we did not consider a design effect in the initial sample size calculation, and that we did not assess the gut microbiome. The latter is a complex and sometimes unrewarding exercise, but could potentially have thrown light on the relationship between hygiene parameters and serum 25OHD concentrations.

## Conclusion

Vitamin D deficiency and insufficiency were highly prevalent in this adult rural south Indian community, with educational status, time of exposure to sunlight, gender, fish consumption and hygiene being factors that independently determined the vitamin D status of individuals. These findings should help in designing and targeting interventions to improve the vitamin D status of the individuals residing in these communities.

## Additional file

Additional file 1: Interview guide / data collection form for the study. Data were collected by the first author through personal interview of each participant using this as the guide. (DOCX 29 kb)

## Abbreviations

25OHD: 25 Hydroxy Vitamin D
EPI: Expanded Programme on Immunization
SES: Socio Economic Status
VDD: Vitamin D Deficiency
