# Supplementary material for: Vitamin D status in Kancheepuram District, Tamil Nadu, India
Source: BMC Public Health. 2018 Dec 5;18:1345. doi: 10.1186/s12889-018-6244-5 (PMC6280507; doi:10.1186/s12889-018-6244-5)
Supplement: Supplementary file 1 — Interview guide / data collection form for the study. Data were collected by the first author through personal interview of each participant using this as the guide. (DOCX 29 kb) [file 12889_2018_6244_MOESM1_ESM.docx]

| **Demographic Information** | | | | | | | | | | | | |
| --- | --- | --- | --- | --- | --- | --- | --- | --- | --- | --- | --- | --- |
| 1. | Name | | |  | | | | | | | | |
| 2. | Age | | |  | | | | | | | | |
| 3. | Gender | | | [1] Male | | | [2] Female | | [3] Transgender | | | |
| 4. | Father / Husband Name | | |  | | | | | | | | |
| 5. | Contact Address | | |  | | | | | | | | |
| 6. | Contact Number | | |  | | | | | | | | |
| 7. | Marital Status | | | [0] Married or Widowed | | | | | [1] Unmarried | | | |
| 8. | Educational Qualification | | | [0] Illiterate | | [2] Primary School | | | [3] High School | | [4] College | |
| 9. | | Occupation | [0] Unemployed | | [1] Skilled Laborer | | | [2] Clerical / Shop owner | | [3] Teacher / Manager | | [4] Professional |
| 10. | Religion | | | [1] Hindu | | [2] Christian | | | [3] Muslim | | [4] Others; Specify______ | |
| 11. | Birth Place | | | [1] Rural (village, hamlet) | | | | | [2] Urban (town, city) | | | |

| **Epidemiological Information** | | | | | |
| --- | --- | --- | --- | --- | --- |
| 12. | What is the source of your drinking water | [1] Bore well | [2] Well | [3] Public tap | [4] Bottled |
| 13. | Do you use filtered or boiled water for drinking? | [0] No | | [1] Yes | |
| 14. | Do you have running tap water in your house? | [0] No | | [1] Yes | |
| 15. | Do you have a closed toilet system at home? | [0] No | | [1] Yes | |
| **Diet habits**  **(Please answer the questions based particularly on your recall of the past two weeks)** | | | | | |
| 16. | Do you drink milk? | [0] No | | [1] Yes | |
| 17. | Do you eat fish such as salmon (Kizhangan meen) or sardine (maththi meen)? | [0] No | | [1] Yes | |
| 18. | Do you have the habit of eating beef? | [0] No | | [1] Yes | |
| 19. | Do you have the habit of eating liver | [0] No | | [1] Yes | |
| **Sun exposure and other information** | | | | | |
| 22. | In the last one month how much time per day on average did you spend outdoors | [1] 15 to 30 minutes  [2] 30 to 60 minutes  [3] 60 to 120 minutes  [4] >120 minutes | | | |
| 23. | In the last one month, what type of dress did you generally wear when going outdoors? | 1. Fully covered (only face and hands exposed) 2. Short sleeved shirt/T-shirt and shorts/dhoti (a single cloth tied around the waist and hitched up to expose the legs) 3. Only shorts or dhoti without shirt or vest | | | |
| 24 | Do you normally use sun screen when going out? | [0] No | | [1] Yes | |
| 25. | Are you using vitamin supplements containing vitamin D? | [0] No | | [1] Yes | |
| 26. | Do you have any awareness of vitamin D (eg. from school, newspapers, radio, TV or health professionals?) | [0] No | | [1] Yes | |

**Socio Economic Status (Modified Kuppuswamy’s Scale 2012)**

**(A) Education of head of the family:**

| **Education** | **Score** |
| --- | --- |
| 1. Profession or Honours | 7 |
| 2. Graduate or Post Graduate | 6 |
| 3. Intermediate or Post High School Diploma | 5 |
| 4. High School Certificate | 4 |
| 5. Middle School Certificate | 3 |
| 6. Primary School Certificate | 2 |
| 7. Illiterate | 1 |

**(B) Occupation of head of the family**

| **Occupation** | **Score** |
| --- | --- |
| 1. Profession (physician, engineer) | 7 |
| 2. Semi-Profession (manager, teacher) | 6 |
| 3. Clerical, Shop-Owner, Farmer | 5 |
| 4. Skilled Worker | 4 |
| 5. Semi-Skilled Worker | 3 |
| 6. Unskilled Worker | 2 |
| 7. Unemployed | 1 |

**(C) Family income**

| **Family Income per Month (in INR) – Original** | **Score** | **Modified for 2012 in (INR)** |
| --- | --- | --- |
| 1. ≥2000 | 12 | ≥32050 |
| 2. 1000 – 1999 | 10 | 16020-32049 |
| 3. 750 – 999 | 6 | 12020-16019 |
| 4. 500 – 749 | 4 | 8010-12019 |
| 5. 300 – 499 | 3 | 4810-8009 |
| 6. 101 – 299 | 2 | 1601-4809 |
| 7. ≤100 | 1 | ≤1600 |

**Total Score**

| **Total Score** | **Socioeconomic Class** |
| --- | --- |
| 26 – 29 | Upper (I) |
| 16 – 25 | Upper Middle (II) |
| 11 – 15 | Lower Middle (III) |
| 5 – 10 | Upper Lower (IV) |
| <5 | Lower (V) |
